# Distinct cytokine patterns may regulate the severity of neonatal asphyxia—an observational study

**DOI:** 10.1186/s12974-017-1023-2

**Published:** 2017-12-12

**Authors:** Anna Bajnok, László Berta, Csaba Orbán, Gábor Veres, Dénes Zádori, Hajnalka Barta, Ünőke Méder, László Vécsei, Tivadar Tulassay, Miklós Szabó, Gergely Toldi

**Affiliations:** 1 First Department of Obstetrics and Gynecology, Semmelweis University, Baross str. 27, Budapest, H-1088 Hungary; 2 First Department of Pediatrics, Semmelweis University, Bókay János str. 53–54, Budapest, H-1083 Hungary; 3 Department of Neurology, Albert Szent-Györgyi Clinical Centre, Faculty of Medicine, University of Szeged, Semmelweis str. 6, 5th floor, Szeged, H-6725 Hungary; 4 MTA-SZTE Neuroscience Research Group, Szeged, Hungary; 5 MTA-SE Pediatrics and Nephrology Research Group, Budapest, Hungary; 6 Birmingham Women’s and Children’s Hospital, Neonatal Unit, Birmingham, UK

**Keywords:** Perinatal asphyxia, Hypoxic ischemic encephalopathy, Neuroinflammation, Cytokine network

## Abstract

**Background:**

Neuroinflammation and a systemic inflammatory reaction are important features of perinatal asphyxia. Neuroinflammation may have dual aspects being a hindrance, but also a significant help in the recovery of the CNS. We aimed to assess intracellular cytokine levels of T-lymphocytes and plasma cytokine levels in moderate and severe asphyxia in order to identify players of the inflammatory response that may influence patient outcome.

**Methods:**

We analyzed the data of 28 term neonates requiring moderate systemic hypothermia in a single-center observational study. Blood samples were collected between 3 and 6 h of life, at 24 h, 72 h, 1 week, and 1 month of life. Neonates were divided into a moderate (*n* = 17) and a severe (*n* = 11) group based on neuroradiological and amplitude-integrated EEG characteristics. Peripheral blood mononuclear cells were assessed with flow cytometry. Cytokine plasma levels were measured using Bioplex immunoassays. Components of the kynurenine pathway were assessed by high-performance liquid chromatography.

**Results:**

The prevalence and extravasation of IL-1b + CD4 cells were higher in severe than in moderate asphyxia at 6 h. Based on Receiver operator curve analysis, the assessment of the prevalence of CD4+ IL-1β+ and CD4+ IL-1β+ CD49d+ cells at 6 h appears to be able to predict the severity of the insult at an early stage in asphyxia. Intracellular levels of TNF-α in CD4 cells were increased at all time points compared to 6 h in both groups. At 1 month, intracellular levels of TNF-α were higher in the severe group. Plasma IL-6 levels were higher at 1 week in the severe group and decreased by 1 month in the moderate group. Intracellular levels of IL-6 peaked at 24 h in both groups. Intracellular TGF-β levels were increased from 24 h onwards in the moderate group.

**Conclusions:**

IL-1β and IL-6 appear to play a key role in the early events of the inflammatory response, while TNF-α seems to be responsible for prolonged neuroinflammation, potentially contributing to a worse outcome. The assessment of the prevalence of CD4+ IL-1β+ and CD4+ IL-1β+ CD49d+ cells at 6 h appears to be able to predict the severity of the insult at an early stage in asphyxia.

## Background

Neonatal asphyxia evokes the injury of the central nervous system (CNS) due to the severe lack of oxygen and perfusion during labor and delivery, resulting in moderate to severe neurological dysfunction. Asphyxia primarily affects term and post-term neonates. It occurs in 2–4 of every 1000 live-born term neonates and is responsible for approximately 23% of neonatal deaths worldwide [1]. While some surviving children show favorable neurological outcome, others sustain severe neurodevelopmental disabilities such as mental retardation, sensory impairment, cerebral palsy, and seizures [2]. Identifying the factors responsible for such extent of individual variability regarding outcome would be critical; however, to date, no definitive predictive factors have been validated.

Inflammation of the CNS, or neuroinflammation, is now recognized to be a feature of all neurological disorders, including that related to neonatal asphyxia. Microglia and astrocytes become activated and release pro-inflammatory cytokines and chemokines. Disruption of the blood-brain barrier allows infiltration of peripheral monocytes into the brain that further enhances the inflammatory response, leading to neuronal injury and apoptosis. However, the inflammatory reaction following asphyxia is not limited to the CNS, but can also be detected in the periphery. Systemic immune activation is characterized by increased synthesis of pro-inflammatory cytokines [3]. A key player in the mediation of the inflammatory response both in the brain and peripheral blood during asphyxia is the subset of T lymphocytes. T lymphocytes have a pivotal role in the evolution of hypoxic injury. The mechanisms by which T cells are neurotoxic include the production of perforin and granzyme B, the release of free radicals, the triggering of apoptotic pathways within neurons, and most importantly, the production of pro- and anti-inflammatory cytokines [4, 5].

An extensive dataset describes neuroinflammation to have detrimental consequences, but results have indicated over the past decade that some aspects of the inflammatory response are beneficial for the CNS outcomes [6, 7]. Benefits of neuroinflammation include neuroprotection, the mobilization of neural precursors for repair, remyelination, and axonal regeneration. In vitro studies demonstrated that pro-inflammatory cytokines, such as TNF-α and IFN-γ, are toxic for oligodendrocytes [8–10]. Although inflammatory cytokines contribute to injury progression, they also play a vital role in the fast elimination of cellular debris, and in the processes of growth and repair, contributing to functional recovery [11, 12]. The results of Saliba et al. support the positive role of certain cytokines in neuronal regeneration [13]. In addition to its toxic effect, TNF-α also plays a role in neuronal progenitor cell proliferation, lineage commitment, and cellular differentiation. IL-1 also has neurotrophic properties which might be mediated by the stimulation of nerve growth factor production. Direct intracerebral injection of IL-1 or TNF-α has been shown to stimulate astrogliosis and angiogenesis in the developing rodent brain [14]. The TGF-β family consists of pleiotropic proteins with potent immunoregulatory properties, which might also play key roles in the development, repair, and survival of neurons [13].

Previous investigations in asphyxia demonstrated that pro-inflammatory IL-1β, TNF-α, and IFN-γ play an outstanding role in the pathophysiology. IL-6, IL-8, and IL-17 (Th17 cells) also have an important contribution [13, 15–17]. On the other hand, anti-inflammatory TGF-β and IL-10 have a protective role and are important for regenerative processes [18]. Prolonged moderate hypothermia improves neurological outcome and has become standard care for term infants with hypoxic-ischemic encephalopathy over the recent years [19]. One mechanism by which hypothermia exerts a neuroprotective effect may be by reducing systemic inflammation [20]. In an earlier study, we measured cytokine levels at the 6th, 12th, and 24th postnatal hours in neonates with asphyxia treated with hypothermia or standard intensive care on normothermia [21]. Our results indicated that IL-6 levels (at 6 h of age) and IL-4 levels (at all time points) were significantly lower in asphyxiated neonates treated with hypothermia compared to normothermic neonates. The duration of hypothermia initiated before 6 h of age correlated with lower levels of IL-6, TNF-α, and IFN-γ measured at 6 h of age. These data suggest that therapeutic hypothermia may rapidly suppress and modify the immediate cytokine response in asphyxia.

The permeability of the BBB is higher in neonates compared to adults and is further disrupted by the hypoxic injury itself. The release of IL-1β, TNF-α, and IFN-γ also increase the permeability of the BBB [22, 23]. CD49d is part of the VLA-4 antigen which mediates the migration of activated leukocytes to the site of tissue inflammation via binding to VCAM-1, expressed by endothelial cells [24]. VLA-4 is thus crucial for the migration of activated T lymphocytes through the BBB to the site of inflammation in the brain [25, 26], making it a primary therapeutic target in multiple sclerosis [27] and in primary neuroinflammatory brain disease in murine ischemic stroke models [28]. Although VCAM-1 is not exclusively expressed in the CNS, the level of CD49d expression can be correlated with the capacity of T lymphocytes to enter the site of inflammation, more specifically the brain tissue in the case of neuroinflammation [29].

The interplay between the kynurenine system and cytokines is a regulator of both innate and adaptive immune responses, and it plays an important role in the interactions between the central nervous and the immune systems [30, 31]. Indoleamine 2,3-dioxygenase (IDO), the rate-limiting enzyme in the degradation of tryptophan, plays a central role in regulating these interactions. IDO degrades TRP to kynurenine (KYN), which is then metabolized by the enzymes within the kynurenine pathway into other catabolites, such as Kynurenic acid (KYNA). While some TRP metabolites may have neurotoxic potential, KYNA appears to be a potent neuroprotective agent as it ameliorates NMDA receptor-mediated excitotoxicity [32] and acts as a potent free radical scavenger and endogenous antioxidant [33]. Induced by pro-inflammatory stimuli (such as IFN-γ), IDO is primarily produced by antigen presenting cells (APCs) and has several immunosuppressive effects, thus maintaining the balance between pro- and anti-inflammatory impulses. The rate of TRP degradation, expressed by the ratio of KYN to TRP (K/T), allows a good estimate of its enzymatic activity [34]. The induction of IDO and the kynurenine system results in the inhibition of T cell functions, the activation of regulatory T cells, and the inhibition of natural killer cells. The alterations of the kynurenine system appear to play a role in the pathophysiology of a broad spectrum of neurological disorders [35], including ischemic brain injury; however, its role has previously not been investigated in perinatal asphyxia.

The challenge in neonatal asphyxia is to harness the beneficial aspects of neuroinflammation following the insult to allow neuroprotection and regeneration within the CNS while at the same time minimizing its harmful effects. Significant barriers remain in understanding the benefits of inflammation in contrast to its detriments following neonatal asphyxia. Identification of factors that differentiate between infants with an extensive and potentially damaging neuroinflammatory response and infants with moderate inflammation would present new options for a more individualized therapeutic approach in neonatal asphyxia. In this study, we aimed to assess the prevalence and cytokine production of T lymphocyte subsets in moderate and severe perinatal asphyxia in order to identify players of the inflammatory response that may influence patient outcome. In contrast to previous studies, we aimed to determine the intracellular cytokine levels of T lymphocytes besides plasma cytokine levels. We also aimed to describe plasma TRP, KYN, and KYNA levels. We also expanded our investigation to 1 month of age following the CNS insult to understand longer-term consequences of the hypoxic event.

## Methods

### Patients

We enrolled 33 term neonates admitted to the regional neonatal intensive care unit at the First Department of Pediatrics at Semmelweis University, Budapest, Hungary, with the initial diagnosis of perinatal asphyxia requiring therapeutic hypothermia. The diagnosis of moderate-to-severe hypoxic-ischemic encephalopathy and the eligibility for cooling was assessed according to the TOBY criteria [36]. Infants fulfilled both criteria A (Apgar score ≤ 5 at 10 min after birth OR continued need for resuscitation, including endotracheal or mask ventilation, at 10 min after birth OR umbilical cord, arterial, or capillary pH < 7.00 within 60 min of birth OR base deficit ≥ 16 mmol/L in umbilical cord or any blood sample (arterial, venous, or capillary) within 60 min of birth) AND criteria B (clinical seizures OR altered state of consciousness (reduced response to stimulation or absent response to stimulation) AND abnormal tone (focal or general hypotonia, or flaccid) AND abnormal primitive reflexes (weak or absent suck or Moro response)). All enrolled neonates were outborn, and hypothermia was initiated between 1 and 5 h of life (mostly within 2 h of life as hypothermia was started before and maintained during transport). Rectal temperature was maintained between 33 and 34 °C and was recorded every hour during the 72 h intervention period. Two-milliliter venous blood samples were collected between 3 and 6 h of life (at admission), as well as at 24 h, 72 h, and 1 week of life during intensive care treatment, adjusted to blood sampling related to clinical care. A further venous blood sample was obtained at 1 month of age during a routine outpatient follow-up appointment.

Neonates with congenital abnormalities or CNS malformations, maternal chorioamnionitis or perinatal infections were excluded from the study. Blood cultures and ear swabs were obtained at admission from all infants, and bacterial infection was excluded. Clinical or culture-proven sepsis was not detected in any of the participating infants. All infants received regular preventive intravenous antibiotics, i.e., ampicillin and gentamicin, during the hypothermic treatment. Four infants were excluded from the analysis as their MRI scan results showed signs of neonatal stroke rather than a hypoxic-ischemic insult. One further infant was excluded due to suspected metabolic disease (peroxisomal fatty acid C26/C22 ratio above the normal range) as well as the presence of multiple minor anomalies and mutation of the ROBO1 gene. Therefore, data from 28 neonates are analyzed in this study. Participating neonates were divided into two groups based on the severity of hypoxic-ischemic insult, determined by initial and recovery time of amplitude-integrated EEG (aEEG) monitoring [37] as well as MRI results performed up to 12 days of life. MRI data were interpreted by radiologists who were blinded to the clinical status of the neonates, based on the criteria defined by Marcovici et al. and Bosmans et al. [38, 39]. The reporting template was developed in the ISORT (intelligent structured online reporting tool) software framework created by Bioscreen Ltd., Debrecen, Hungary. In cases where MRI was not performed due to the critical condition of the patient or MRI data were missing, grouping was done solely based on the aEEG results. The severe group (*n* = 11) consisted of newborns with moderate-to-severe hypoxic-ischemic encephalopathy (HIE) signs on MRI scans AND burst-suppression or continuous extremely low voltage or flat tracing background activity on aEEG OR normalization of aEEG after the 48th hour of life or never, OR early death (< 28 days). Neonates that met none of the above-listed criteria constituted the moderate group (*n* = 17) (normal MRI scans or mild HIE signs on MRI scans AND continuous or discontinuous normal voltage background activity on aEEG OR normalization of aEEG activity before the 48th hour of life).

In the severe group, three infants deceased before 1 month of age due to the severity of the insult. Available data from these neonates were included at the relevant time points within the severe group. Therefore, 72-h, 1-week, and 1-month data were missing in case of two infants, and 1-month data were missing from one infant.

Our study was reviewed and approved by the Hungarian Medical Research Council (TUKEB 6578-0/2011-EKU), and written informed consent was obtained from parents of all participants. The study was adhered to the tenets of the most recent revision of the Declaration of Helsinki. Clinical characteristics and laboratory parameters of participants are summarized in Table 1.

**Table 1:** Clinical characteristics of neonates in the moderate and severe groups upon admission (within 12 h of age)

	Moderate group (*n* = 17)	Severe group (*n* = 11)
Male gender (%)	10 (59)	7 (64)
Birthweight (g)	3330 (2860–3605)	3000 (2490–3300)
Gestational age (week)	39 (37–40)	38 (37–40)
No. of C-sections (%)	10 (59)	8 (73)
Apgar at 1 min	3 (0.5–4.5)	1 (0–3)
Apgar at 5 min	6 (5–7)	2* (0–4)
Apgar at 10 min	7 (5–8)	4* (1.75–5.25)
Worst pH (within first 12 h)	7.025 (6.874–7.12)	6.86 (6.62–7.058)
Worst BD (within first 12 h) (mmol/L)	18.05 (16.65–21.275)	20.4 (19.375–23.5)
Need for inotropes (%)	8 (44)	8* (73)
Need for hydrocortisone (%)	5 (36)	5 (45)
Need for erythrocyte transfusion (%)	7 (41)	4 (36)
Need for platelet transfusion (%)	2 (11)	1 (9)
Need for fresh frozen plasma (%)	6 (35)	10* (91)
S100 (ug/L)	7.48 (2.33–28.85)	21.8 (3.8–30.0)
LDH (U/L)	2072 (1371–5274)	3335 (1879–5792)
Uric acid (umol/L)	530 (474.5–576.5)	593 (443–634)
AST (U/L)	112 (79–363)	207 (128–438)
ALT (U/L)	24 (19–125)	54 (29–148)
GGT (U/L)	123 (73–198)	97 (47–197)
Urea (mmol/L)	4.9 (3.7–5.3)	4.8 (3.9–5.7)
Creatinine (umol/L)	88 (78.5–99)	105 (80–114)

Data are presented as median (IQR)

*p < 0.05 vs Moderate group

### Flow cytometry

Plasma was separated from peripheral blood samples by centrifugation. Plasma samples were aliquotted and immediately frozen and stored at − 80 °C for later determination of cytokine concentrations and HPLC measurements.

Remaining cells were resuspended in RPMI (Roswell Park Memorial Institute)-1640 medium (Sigma-Aldrich, St. Louis, MO, USA). Cells were incubated with PMA (phorbol 12-myristate 13-acetate) (50 ng/ml), ionomycin (1 μg/mL), and BFA (brefeldin A) (10 μg/ml) for 6 h at 37 °C to allow intracellular accumulation of cytokines. For surface marker staining, samples were then incubated with the following fluorochrome-conjugated anti-human monoclonal antibodies: CD4 PE-Cy7 (phycoerythrin-cyanine 7) and CD8 APC-Cy7 (allophycocyanin-cyanine 7) (panel 1) or CD4 APC-Cy7 and CD49d PerCP (peridinin-chlorophyll-protein) (panel 2), respectively, according to the manufacturers’ instructions (all from BioLegend, San Diego, CA, USA). Red blood cells were lysed and PBMCs were permeabilized using FACSLysing and FACSPermeabilizing solutions (BD Biosciences, San Jose, CA, USA). Cells were washed and resuspended in PBS (phosphate buffer saline) and divided into two equal aliquots and stained according to the manufacturers’ instructions for intracellular cytokines using the following conjugated anti-human monoclonal antibodies or the appropriate isotype controls: IL-6 PE (phycoerythrin), IL-17A PerCP, IL-10 APC (allophycocyanin), and IFN-γ FITC (fluorescein isothiocyanate) (for panel 1) or TNF-α PE-Cy7, FoxP3 PE, TGF-β APC, and IL-1β FITC (for panel 2), respectively (all from BioLegend). Following labeling, cells were washed and resuspended in PBS for flow cytometry analysis. Samples were analyzed immediately on a FACSAria flow cytometer (BD Biosciences) equipped with 488 and 633 nm excitation lasers. Data were processed using the FACSDiVa software (BD Biosciences). One hundred thousand cells were recorded. Evaluators of flow cytometry data were blinded to the clinical status of the neonates.

### Immunoassays

Plasma samples were stored at − 80 °C until analysis. The plasma levels of the following cytokines, chemokines, and growth factors were determined using Bio-Plex Pro Assays (Bio-Rad Laboratories, Hercules, CA, USA): IL-1b, IL-2, IL-4, IL-5, IL-6, IL-7, IL-8, IL-10, IL-12, IL-13, IL-17, IFN-γ, TNF-α, TGF-β, G-CSF, GM-CSF, MCP-1, MIP-1b, and VCAM. Bio-Plex Pro Assays are immunoassays formatted on magnetic beads that utilize principles similar to those of a sandwich ELISA. Capture antibodies against the biomarker of interest are covalently coupled to the beads. A biotinylated detection antibody creates the sandwich complex, and the final detection complex is formed by the addition of a streptavidin-phycoerythrin (SA-PE) conjugate, where PE serves as the fluorescent reporter. Reactions are read using a Luminex-based reader.

### High-performance liquid chromatography

Plasma samples were stored at − 80 °C until analysis. Directly prior to analysis, samples were thawed, vortexed, and 300 μl of plasma was “shot” onto 700 μl of precipitation solvent (containing 3.57 *w*/*w*% perchloric acid and 2.857 mM 3-nitro-l-tyrosine as internal standard (Scharlau, Barcelona, Spain)). Samples were then centrifuged (13,000 *g* for 10 min at 4 °C), and the supernatants were collected. For the quantification of KYN, KYNA, and TRP concentrations of samples, a modified method based on Herve et al. [40] was utilized, using an Agilent 1100 HPLC system (Agilent Technologies, Santa Clara, CA, USA). The system was equipped with a fluorescent detector, which was used to determine the concentration of KYNA and TRP, and a UV detector which was applied for the determination of KYN and the internal standard. Chromatographic separations were performed on an Onyx monolithic C18 column, 100 × 4.6 mm I.D. (Phenomenex Inc., Torrance, CA, USA) after passage through a Hypersil ODS precolumn, 20 × 2.1 mm I.D., 5-μm particle size (Agilent Technologies) with a mobile phase composition of 0.2 M zinc acetate/acetonitrile 95/5 *v*/*v*% with a pH adjusted to 6.2 with glacial acetic acid, applying isocratic elution. The flow rate and the injection volume were 1.5 mL/min and 20 μL, respectively. The fluorescent detector was set at excitation and emission wavelengths of 344 and 398 nm, and after 3.5 min of each run, the wavelengths were changed to 254 and 398 nm. The UV detector was set at a wavelength of 365 nm. L-TRP, L-KYN sulfate salt, KYNA, and zinc acetate dihydrate were purchased from Sigma-Aldrich, and acetic acid was purchased from VWR International (Radnor, PA, USA).

### Statistical analysis

Data are expressed as median and interquartile range. Comparisons between sample populations were performed with Mann-Whitney tests, as a test of normality (performed according to Kolmogorov-Smirnoff) indicated the non-normal distribution of data. Comparisons between the paired values (samples collected at different time points) in the same population were made with Friedman tests. *p* values less than 0.05 were considered significant. Outliers were identified using Grubbs’ tests and were excluded from the analyses. Statistics were calculated using the GraphPad Prism 5 software (La Jolla, CA, USA).

## Results

### IL-1β

**Fig. 1:**
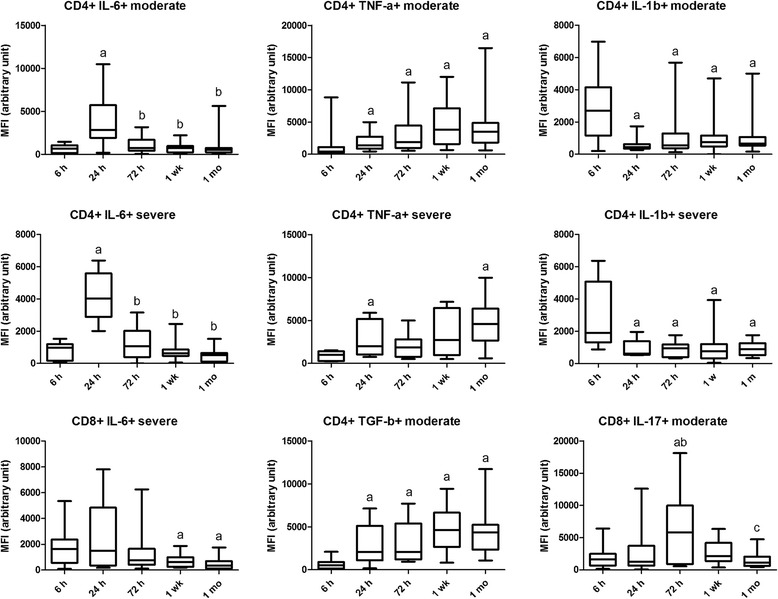
Intracellular cytokine level alterations in time represented by mean fluorescence intensity (MFI) values in moderate and severe asphyxia. Horizontal line, median; box, interquartile range; whisker, range. *p* < 0.05 a vs 6 h, b vs 24 h, c vs 72 h, d vs 1 week

**Fig. 2:**
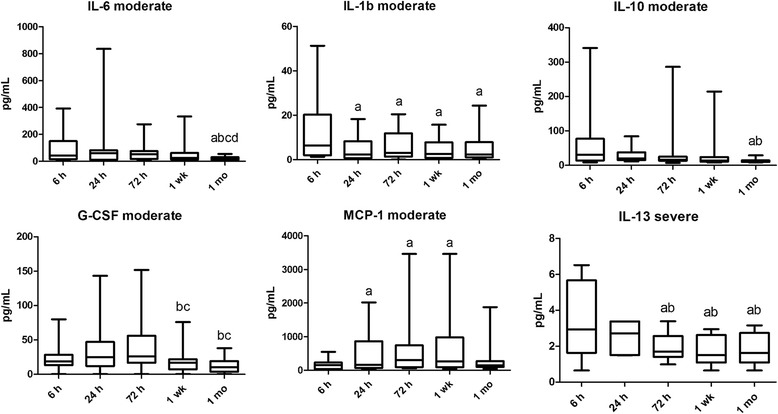
Plasma cytokine level alterations in time in moderate and severe asphyxia. Horizontal line, median; box, interquartile range; whisker, range. *p* < 0.05 a vs 6 h, b vs 24 h, c vs 72 h, d vs 1 week

Our results suggest that CD4+ IL-1β+ cells are early mediators of the inflammatory response, as their prevalence is higher at 6 h after birth in severe compared to moderate asphyxia. The extravasation of these cells is also increased at this time point in severe asphyxia as evidenced by the lower prevalence of CD49d-expressing CD4+ IL-1β+ cells in peripheral blood. Therefore, although plasma levels of IL-1β are not different in moderate and severe asphyxia, IL-1β may play an important role in initiating tissue damage in the brain following the hypoxic insult. Intracellular (MFI) levels of IL-1β in both groups and plasma levels in the moderate group are highest at 6 h and comparably lower at the following time points, suggesting that its main role is the initiation of the inflammatory response (Table 2 and Figs. 1 and 2).

**Table 2:** Significant differences in intracellular cytokine, plasma cytokine, and high-performance liquid chromatography (HPLC) data between the moderate and severe group

	Time	Moderate	Severe
Intracellular cytokines—cell prevalence data (% of parent population)			
CD4+ IL-1b+/CD4+	6 h	3.52 (2.13–5.16)	6.77 (3.18–10.26)
CD4+ IL-1b+ CD49d+/CD4+ IL-1b+	6 h	6.98 (4.61–9.32)	4.08 (2.86–5.46)
CD4+ TNF-a + CD49d+/CD4+ TNF-a+	6 h	6.63 (4.47–13.45)	3.52 (2.12–7.23)
CD8+ IL-17+/CD8+	6 h	5.26 (3.89–14.40)	2.63 (1.75–5.18)
CD4+ FoxP3+/CD4+	24 h	2.35 (1.96–3.13)	3.02 (2.60–4.13)
CD4+ TNF-a+ CD49d+/CD4+ TNF-a+	72 h	4.77 (3.43–7.70)	9.75 (6.31–10.80)
CD4+ IL-17+/CD4+	1 week	3.08 (1.80–4.59)	5.13 (3.40–13.76)
Intracellular cytokines—mean fluorescence intensity (MFI) data (arbitrary unit)			
CD8+ IL-17+/CD8+	24 h	1069 (639–3265)	4187 (1274–6133)
CD4+ IFN-g+/CD4+	72 h	455 (150–770)	887 (496–1427)
CD4+ IL-17+/CD4+	72 h	939 (566–1674)	1760 (1614–3508)
CD4+ TNF-a+/CD4+	1 month	3281 (1752–4326)	4729 (3959–6714)
Plasma cytokines (pg/mL)			
G-CSF	24 h	19.85 (10.87–30.70)	42.74 (22.27–131.3)
IL-5	72 h	1.37 (0.00–4.69)	0.20 (0.00–0.46)
IL-13	72 h	2.35 (2.01–3.67)	1.70 (1.40–2.56)
IL-6	1 week	21.06 (11.89–43.24)	70.25 (33.73–134.1)
G-CSF	1 week	13.33 (5.52–17.72)	32.90 (16.65–94.76)
HPLC results (uM)			
KYN	1 month	3.62 (2.72–4.47)	2.28 (1.45–3.14)

*p* < 0.05 for all comparisons

### IL-6

Plasma IL-6 levels were higher at 1 week in the severe compared with the moderate group. Plasma IL-6 levels decreased by 1 month following the insult in the moderate group. Mean fluorescence intensity (MFI) of CD4+ IL-6+ cells peaked at 24 h in both patient groups and declined later, indicating that it may play a role in the initial inflammatory response. MFI of CD8+ IL-6+ cells in the severe group also decreased by 1 week (Table 2 and Figs. 1 and 2).

### IL-17

**Fig. 3:**
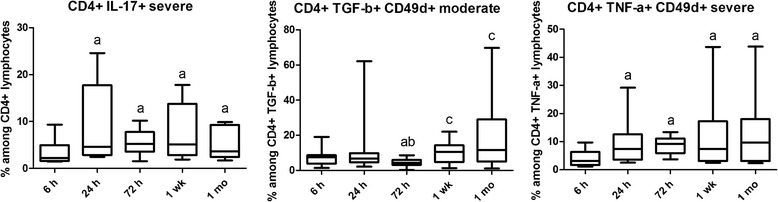
Cell prevalence data alterations in time in moderate and severe asphyxia. Horizontal line, median; box, interquartile range; whisker, range. *p* < 0.05 a vs 6 h, b vs 24 h, c vs 72 h, d vs 1 week

The prevalence of CD8+ IL-17+ cells was higher in the moderate group than in the severe group at 6 h. In contrast, the prevalence of CD4+ IL-17+ cells was lower in the moderate than in the severe group at 1 week The prevalence of CD4+ IL-17+ was lower at 6 h than at other time points in the severe group and remained high until 1 month. MFI of CD8+ IL-17+ cells at 24 h and that of CD4+ IL-17+ cells at 72 h were also higher in the severe group. The MFI of CD8+ IL-17+ cells peaked at 72 h in the moderate group. No difference was observed in plasma levels of IL-17 (Table 2 and Figs. 1 and 3).

### TNF-α

On the contrary, MFI of TNF-α in CD4 cells was increased at all time points compared to 6 h in both groups. At 1 month, MFI of TNF-α was higher in the severe group, suggesting that it might play a role in the development of long-term consequences of asphyxia. The prevalence of CD49d-expressing CD4+ TNF-α+ cells is lower in severe asphyxia at 6 h compared to later time points, indicating that in a severe insult, it might also contribute to early tissue destruction through increased extravasation. This is further supported by the fact that the prevalence of CD49d-expressing CD4+ TNF-α+ cells is lower at 6 h and higher at 72 h in severe asphyxia compared to a moderate insult. No difference was observed in plasma levels of TNF-α (Table 2 and Figs. 1 and 3).

### Other pro-inflammatory cytokines

MFI of CD4+ IFN-γ+ cells was higher in the severe than in the moderate group at 72 h. Plasma MCP-1 levels were higher at 24 and 72 h as well as 1 week than at 6 h in the moderate group. Plasma G-CSF levels were higher at 24 h and 1 week in the severe compared with the moderate group. G-CSF levels decreased by 1 week and remained low at 1 month following the initial insult in the moderate group (Table 2 and Fig. 2).

### TGF-β

The prevalence of CD49d-expressing CD4+ TGF-β+ cells was increased at 1 week and 1 month compared to 72 h in the moderate group potentially indicating that TGF-β plays an anti-inflammatory role in tissue regeneration in the early stage of the insult. MFI of CD4+ TGF-β+ cells was increased from 24 h onwards in the moderate but not in the severe group, which is probably also part of a compensatory phenomenon (Figs. 1 and 3).

### Other anti-inflammatory cytokines

Plasma IL-10 levels were lower at 1 month than at 6 and 24 h in the moderate group. Plasma IL-13 and IL-5 levels were higher in the moderate than in the severe group at 72 h. Plasma IL-13 levels were higher at 6 and 24 h than the following time points in the severe group (Table 2 and Fig. 2).

### Tregs

The prevalence of Tregs is somewhat higher in severe asphyxia at 24 h, which might be part of a compensatory mechanism; however, the biological significance of this increase is questionable (Table 2).

### The kynurenine system

**Fig. 4:**
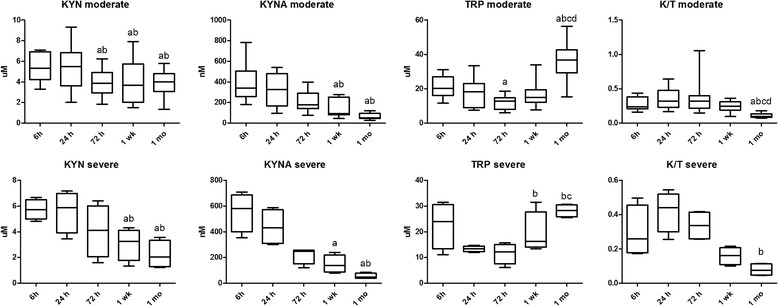
Alterations in the components of the kynurenine pathway in time in moderate and severe asphyxia. Horizontal line, median; box, interquartile range; whisker, range. *KYN* kynurenine, *KYNA* kynurenic acid, *TRP* tryptophan, *K/T* kynurenine/tryptophan ratio. *p* < 0.05 a vs 6 h, b vs 24 h, c vs 72 h, d vs 1 week

Plasma KYN levels were higher at 1 month in the moderate group compared to the severe group. KYN levels showed a decline in both groups by 1 week and 1 month following the insult. Similar results were observed for KYNA, while TRP levels increased significantly by 1 month in both groups. In line with the above, the K/T ratio, corresponding to the enzymatic activity of IDO, plummeted by 1 month in both groups (Table 2 and Fig. 4).

### ROC analysis

**Fig. 5:**
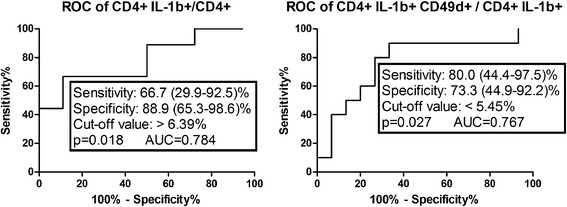
Receiver operator curve (ROC) analysis of the prevalence of CD4+ IL-1β+ and CD4+ IL-1β + CD49d+ cell subsets in moderate and severe neonatal asphyxia. *AUC* area under the curve

We performed ROC analyses to assess which parameters have the potential to discriminate between a moderate and a severe insult at an early stage. The only significant results of the ROC analyses were related to intracellular IL-1β. The prevalence of CD4+ IL-1β+ cells at 6 h (*p* = 0.018, ROC AUC = 0.784) and that of CD4+ IL-1β+ CD49d+ cells at 6 h (*p* = 0.027, ROC AUC = 0.767) was able to differentiate severity with a reasonable sensitivity and specificity (Fig. 5).

## Discussion

Several studies have described the importance of cytokines in normal neuronal differentiation and survival [41–43]. The perinatal brain might be particularly susceptible to alterations in cytokine concentrations, and experimental data suggest that cytokines play a pivotal role in the regulatory network orchestrating neuroinflammation [44]. Cytokines have been in the limelight of research focusing on asphyxia. The inflammatory response following hypoxic brain injury has been shown to have dual effects. A certain level of inflammation appears to be necessary for the adequate regeneration of the brain tissue [6], while extensive neuroinflammation might contribute to further CNS injury and be an important factor in worse functional outcome. We aimed therefore to determine factors that might differentiate between infants who have an adequate level of inflammatory response which is necessary for neuroregeneration from infants in whom the uncompensated inflammatory response contributes to brain injury. Previous studies have focused on determining the level of cytokines from plasma. However, plasma cytokine levels have been shown to have a great variability compared with intracellular cytokine levels, which closely reflect cytokine production at a cellular level and show more stable kinetics in time, and thus open new possibilities for more precise characterization of the cytokine network in immune disorders. The relationship between cellular cytokine production and serum cytokine levels is undefined; cytokines in the serum originate from various different sources and show less stable kinetics. The advantage of intracellular cytokine analysis by flow cytometry is that with this method, the cytokine production of each cell type can be accurately described. This method opens up the opportunity for precise characterization of the function of each cell type in a physiological setting (i.e., maintaining autocrine and cell-cell interactions), which could be of great value in identifying key cellular players of various inflammatory conditions [45, 46]. We therefore primarily aimed to describe intracellular cytokine values; however, we also measured cytokine levels from plasma to gain a more comprehensive picture of the immunologic alterations following the hypoxic insult. Furthermore, contrary to previous studies, we expanded sampling of infants to 1 month of age to obtain data on longer-term changes in inflammatory parameters evoked by asphyxia.

### IL-1β

IL-1β is an important mediator of pro-inflammatory responses [47] and has been reported to have neurotoxic properties leading to BBB breakdown and apoptotic neuronal death [48, 49]. Aly et al. found that the CSF level of IL-1β in term neonates had the highest predictive value of poor neurologic outcome in asphyxia after 6 and 12 months and suggested the central role of IL-1β in the ongoing neuronal injury that occurs in the latent phase following the original hypoxic insult [16]. They also found a high CSF to plasma ratio of IL-1β, indicating elevated local production of the cytokine in the CNS. Several animal models also suggest that IL-1β contributes to the brain injury [50, 51]. The exacerbation of ischemic brain injury has also been observed following exogenous administration of IL-1β. Other studies demonstrated that the deficiency of IL-1β converting enzyme or treatment with IL-1 receptor antagonists (IL-1ra) resulted in the moderation of hypoxic brain injury [52, 53], decreased post-ischemic edema [49], and improved neurological outcome [54].

The primary sources of IL-1β are APCs and monocytes [55, 56], although microglia and endothelial cells are also capable of producing IL-1β [57, 58]. The fact that T lymphocytes are able to produce physiologically relevant amounts of IL-1β and that it plays an important role in their functionality has only recently been revealed [59]. In this study, we observed significant IL-1β production in T lymphocytes in neonatal asphyxia, which was more pronounced in a severe insult. In line with the previous findings [16], our current results suggest that CD4+ IL-1β+ cells might play an important role in initiating tissue damage in the brain following the hypoxic insult. However, intracellular concentrations of IL-1β gradually decreased in both patient groups, suggesting that a certain level of initial increase may be necessary for the regenerative processes as well. Based on our ROC analysis, the prevalence of IL-1β-producing CD4+ T cells may be useful in the differentiation of the severity of the insult at an early stage, up to 6 h after birth (Fig. 5). The notable amount of data showing the therapeutic benefits of peripheral administration of IL-1ra following ischemic brain injury [60] and the fact that IL-1 receptor antagonistic agents are already available in clinical use in autoimmune disorders prompts further research to explore whether IL-1β levels above a certain threshold may be a potential future therapeutic target in neonatal asphyxia.

### IL-6

Several previous studies have associated elevated IL-6 CSF levels with poor neurological outcome, cerebral palsy, and death in asphyxia [44, 61]. However, Aly et al. suggested that IL-6 might have neurotrophic as well as neuroprotective, anti-inflammatory effects via inhibiting the synthesis of TNF-α and IL-1β [16, 62]. They found highly elevated IL-6 CSF to plasma ratios, and Martin-Ancel et al. also concluded that IL-6 appears to be primarily produced intrathecally following the ischemic brain injury while diffusion from the plasma is secondary [44]. In this study, we found that plasma IL-6 levels were elevated in severe compared to moderate asphyxia at 1 week and decreased in moderate, but not in severe asphyxia by 1 month. Intracellular levels of IL-6 in CD4+ cells peaked at 24 h in both patient groups and declined later. However, we found no alterations in intracellular cytokine levels or cellular prevalence data between the two study groups, suggesting that CSF levels of IL-6 might be of more importance with regard to its deleterious effects.

### IL-17

IL-17 is a pro-inflammatory cytokine produced primarily by Th17 cells upon IL-23 stimulation [63]. IL-17 has been shown to play a pivotal role in the delayed progression of brain infarction following hypoxic injury in a mouse brain ischemia model. This is further supported by the fact that IL-17KO mice show a significant reduction in the infarcted area and apoptotic neuronal death from the fourth post-stroke day onwards [64]. Yang et al. reported a significant influx of Th17 cells into the brain tissue in hypoxic-ischemic encephalopathy following LPS (lipopolysaccharide)-sensitization both in neonates and in newborn rats [65]. In line with the previous findings in mouse models, we observed a delayed increase in the prevalence and cytokine production of IL-17 producing T cells, which remained elevated in the severe group during the whole observation period. The prevalence of Th17 lymphocytes was higher in the severe group than in the moderate group at 1 week. IL-17 could play an important role in maintaining the chronic neuroinflammation leading to detrimental consequences.

### TNF-α

TNF-α is a pro-inflammatory cytokine which stimulates the production of IL-1β and among other cytokines regulates the apoptosis of CNS cells, promotes leukocyte differentiation, proliferation, and subsequent CNS infiltration [66, 67]. There is an extensive amount of data supporting the role of TNF-α in ischemic brain damage [52, 68–71]. The level of TNF-α in the CNS has been shown to peak 6–12 h following the hypoxic-ischemic injury in newborn rats [61]. Increased TNF-α and IL-1β plasma and CSF levels in term infants with asphyxia have been associated with neuroradiological alterations, poor neurological status at 12 months of age, and cerebral palsy [72, 73]. Blocking TNF-α, for example, by the administration of pentoxifylline, a competitive inhibitor of TNF-α, improved neurological outcome by attenuating ICAM-1 expression, reducing the disruption of the BBB and protecting neurons from delayed cell death in animal models of head trauma [74].

In line with the previous findings, we found that the MFI of TNF-α in CD4 cells was increased in both groups at all time points compared to 6 h, suggesting a delayed increase in production of TNF-α by T cells following the insult. In severe asphyxia, we observed higher MFI of TNF-α at 1 month than in moderate asphyxia, which might indicate that TNF-α plays a role in maintaining a chronic inflammatory response in severe asphyxia, thus contributing to long-term consequences. We found increased extravasation of TNF-α producing cells at 6 h (indicated by the decreased prevalence of CD49d-expressing CD4+ TNF-α+ cells), which may indicate the role of TNF-α in determining the extent of the initial tissue injury as well. At later time points, we found that the expression of CD49d increased on TNF-α-producing CD4 cells in severe asphyxia, which might further indicate the increased potential of these cells to enter the CNS. Rothhammer et al. were able to demonstrate that under Th1 differentiation promoting circumstances, naive T cells (CD4+ CD44− FoxP3−) differentiate into encephalitogenic T cells in approximately 3 days, expressing high amounts of CD49d in mice [75]. It is, therefore, possible that an initial decrease in the prevalence of CD49d+ lymphocytes due to extravasation is followed by differentiation of CD4+ CD49d+ lymphocytes from the naïve T lymphocyte pool, leading to an increased prevalence of circulating CD49d+ cells as part of an ongoing inflammatory response.

### Anti-inflammatory factors

In order to comprehensively assess the immunosuppressive components of the adaptive immune system in neonatal asphyxia, we examined TGF-β levels, along with the prevalence of Treg cells and the involvement of the KYN pathway. TGF-β plays a critical role in immunosuppression both by inhibiting inflammatory cells and promoting the function of Treg cells via inducing their FoxP3 expression [76–82]. Activated Tregs then produce large amounts of TGF-β, which acts as an important autocrine signal in their activation [83]. TGF-β specifically limits Th1 differentiation and expansion [84, 85] without affecting Th2 effector function and suppresses the production of pro-inflammatory cytokines, while promoting the production of anti-inflammatory IL-10 [86]. Besides direct inhibition, Tregs also inhibit T cell function by affecting the APC-T cell interactions, for example via the CTLA-4 engagement-induced TRP catabolism by IDO [87, 88].

TGF-β is associated with the reparation of the infarcted tissue and thus is expressed later than pro-inflammatory cytokines [13]. Interestingly, we observed an elevation after 24 h in the intracellular level of TGF-β in moderate asphyxia that was not present following a severe insult, where the level of TGF-β remained comparable to the 6 h level throughout the whole observation period. We found increased CD49d-expression, which indicates a higher potential of TGF-β producing cells to enter the CNS at 1 week and 1 month compared to 72 h in the moderate group. These findings suggest that TGF-β plays an important role in attenuating the inflammatory response and in tissue regeneration following the hypoxic insult in moderate asphyxia. The lack of this effect may contribute to a more severe outcome. We found a moderately elevated prevalence of Tregs in severe compared to moderate asphyxia at 24 h, which might be part of a compensatory mechanism; however, the biological significance of this increase is unremarkable.

Plasma KYN levels were higher at 1 month in the moderate than in the severe group, which might contribute to an immunosuppressive effect. KYN levels showed a decline in both groups by 1 week following the insult (Fig. 4). Similar results were observed for KYNA, while TRP levels increased significantly by 1 month in both groups. In line with the above, the K/T ratio, indicating IDO enzymatic activity, plummeted by 1 month in both groups. This increased activation of IDO and TRP catabolism in the postnatal period (up to 1 week) appears to be part of a regulatory mechanism that might play an important role in attenuating the inflammatory response following the hypoxic insult. This effect was comparable in moderate and severe asphyxia, which could mean that this early activation of the KYN pathway is part of the physiological process that accompanies the neuroinflammatory response. However, it appears that the importance of this regulatory mechanism decreases by 1 month.

A limitation of our study is that we did not investigate cell prevalence or cytokine levels in CSF samples. Although this would have provided further data on the local inflammatory response in the CNS, the collection of CSF samples was not possible due to ethical considerations in the lack of clinical indication. A further limitation of this study is the fact that five neonates in each study group (Table 1) received hydrocortisone during intensive care which might have influenced their cytokine balance. Further studies are needed to establish the immunologic effects of hydrocortisone therapy in HIE infants undergoing intensive therapy. Finally, the relatively low number of participants limits the direct clinical utility of the assessment of the prevalence of CD4+ IL-1β+ and CD4+ IL-1β+ CD49d+ cells at 6 h as a predictive marker for the severity of the insult at an early stage in asphyxia.

## Conclusion

In conclusion, the need for more specific prognostic markers other than clinical assessment in neonatal asphyxia is clear, since clinical signs often do not correlate with neurological outcome and do not enable differentiation between moderate and severe hypoxic-ischemic encephalopathy. The role of various cytokines in neuroinflammation following hypoxic-ischemic injury is supported by a rapidly expanding body of evidence. IL-1β and IL-6 appear to play a key role in the early events of the inflammatory response, while TNF-α seems to be responsible for triggering a prolonged inflammation, potentially contributing to a worse outcome. On the other hand, TGF-β has a compensatory role in decreasing the level of inflammation from an early stage following the insult (Table 3). Based on ROC analysis, the assessment of the prevalence of CD4+ IL-1β+ and CD4+ IL-1β+ CD49d+ cells at 6 h appears to be able to predict severity at an early stage in asphyxia (Fig. 5). Our current results open a potentially fruitful area of research as well as diagnostic and therapeutic development in neonatal asphyxia.

**Table 3:** Summary of the proposed effects of distinct cytokines on the severity of neonatal asphyxia

	Pro-inflammatory	Anti-inflammatory
Contribution to better outcome	IL-1β: rapid decrease, higher initial prevalence, and extravasation in severe insult G-CSF: rapid decrease in moderate insult, higher plasma levels in severe insult	TGF-β: increased production and extravasation in moderate insult IDO: early compensation up to 1 week
Contribution to worse outcome	TNF-α: elevated intracellular levels up to 1 month IL-17: high prevalence in severe insult up to 1 month IL-6: higher plasma levels in severe insult at 1 week, decrease in moderate insult by 1 month	Treg: unremarkable difference at 24 h, not upregulated

## Abbreviations

APC: Antigen presenting cell
BBB: Blood-brain barrier
CD: Cluster of differentiation
CTLA-4: Cytotoxic T-lymphocyte associated protein 4
IDO: Indoleamine 2,3-dioxygenase
IFN-γ: Interferon-γ
IL: Interleukin
KYN: Kynurenine
KYNA: Kynurenic acid
MFI: Mean fluorescence intensity
NMDA: *N*-methyl-d-aspartate
PBMC: Peripheral blood mononuclear cells
TGF-β: Transforming growth factor-β
Th: Helper T lymphocyte
TNF-α: Tumor necrosis factor-α
Treg: Regulatory T lymphocyte
TRP: Tryptophan
VCAM-1: Vascular cell adhesion molecule-1
VLA-4: Very Late Antigen-4
